# A pharmacological screen for compounds that rescue the developmental lethality of a Drosophila *ATM* mutant

**DOI:** 10.1371/journal.pone.0190821

**Published:** 2018-01-16

**Authors:** Stacey A. Rimkus, David A. Wassarman

**Affiliations:** Department of Medical Genetics, School of Medicine and Public Health, University of Wisconsin-Madison, Madison, WI; National Center for Geriatrics and Gerontology, JAPAN

## Abstract

Ataxia-telangiectasia (A-T) is a neurodegenerative disease caused by mutation of the *A-T mutated* (*ATM*) gene. *ATM* encodes a protein kinase that is activated by DNA damage and phosphorylates many proteins, including those involved in DNA repair, cell cycle control, and apoptosis. Characteristic biological and molecular functions of ATM observed in mammals are conserved in *Drosophila melanogaster*. As an example, conditional loss-of-function *ATM* alleles in flies cause progressive neurodegeneration through activation of the innate immune response. However, unlike in mammals, null alleles of *ATM* in flies cause lethality during development. With the goals of understanding biological and molecular roles of ATM in a whole animal and identifying candidate therapeutics for A-T, we performed a screen of 2400 compounds, including FDA-approved drugs, natural products, and bioactive compounds, for modifiers of the developmental lethality caused by a temperature-sensitive *ATM* allele (*ATM*^*8*^) that has reduced kinase activity at non-permissive temperatures. Ten compounds reproducibly suppressed the developmental lethality of *ATM*^*8*^ flies, including Ronnel, which is an organophosphate. Ronnel and other suppressor compounds are known to cause mitochondrial dysfunction or to inhibit the enzyme acetylcholinesterase, which controls the levels of the neurotransmitter acetylcholine, suggesting that detrimental consequences of reduced ATM kinase activity can be rescued by inhibiting the function of mitochondria or increasing acetylcholine levels. We carried out further studies of Ronnel because, unlike the other compounds that suppressed the developmental lethality of homozygous *ATM*^*8*^ flies, Ronnel was toxic to the development of heterozygous *ATM*^*8*^ flies. Ronnel did not affect the innate immune response of *ATM*^*8*^ flies, and it further increased the already high levels of DNA damage in brains of *ATM*^*8*^ flies, but its effects were not harmful to the lifespan of rescued *ATM*^*8*^ flies. These results provide new leads for understanding the biological and molecular roles of ATM and for the treatment of A-T.

## Introduction

Ataxia-telangiectasia (A-T) is an autosomal recessive disorder characterized by progressive cerebellar atrophy, immunodeficiency, and cancer predisposition [1–3]. The cause of A-T is mutation of the *A-T Mutated* (*ATM*) gene, which encodes a phosphatidylinositol-3-kinase (PI3K)-related protein kinase involved in the recognition and repair of DNA damage. Loss of *ATM* function leads to a variety of cellular and molecular abnormalities, including accumulation of DNA damage, oxidative stress, insulin resistance, mitochondrial dysfunction, cell cycle dysregulation, and neurodegeneration. Currently, no specific treatment is available for A-T, but studies of *ATM*-deficient cells and animals are making progress toward this goal. High-throughput screens have identified compounds that induce translational read-through of nonsense mutations in *ATM* [4]). In addition, large-scale proteomic, metabolomic, and transcriptomic studies have identified potential cellular therapeutic targets [5]. Lastly, directed approaches have identified possible therapeutics for specific physiological manifestations of A-T; antioxidants reduce oxidative stress [6], glutamine supplementation blocks neuronal cell cycle reentry and improves the DNA damage response [7], and increasing intracellular NAD+ promotes the elimination of dysfunctional mitochondria by mitophagy, a selective form of autophagy [8]. Nevertheless, an opportunity that is yet to be explored because of issues of cost and feasibility is an unbiased screen for compounds that improve *ATM* mutant phenotypes in a whole animal.

Screens of libraries of compounds in *Drosophila melanogaster* have identified candidate therapeutics for diverse human diseases, including Fragile X syndrome [9], thyroid cancer [10], and Alexander disease [11]. Medium-throughput screens of compounds are possible in flies because flies have a short lifecycle, are maintained in small vials, and feed on inexpensive food, which collectively make it economical and feasible to screen large numbers of animals. Flies also have a short lifespan, which allows age-related phenotypes to be analyzed in a reasonable period of time. Lastly, compounds can be easily administered to flies in food and are only needed in small quantities.

*ATM* mutant flies exhibit phenotypes similar to those in A-T, including DNA damage, sensitivity to ionizing radiation, and progressive neurodegeneration [12–17]. A key difference between flies and mammals is that *ATM* is essential in flies. The essential nature of *ATM* in flies may be due to the fact that flies lack the catalytic subunit of DNA-dependent protein kinase (DNA-PK(cs)), which like ATM is a PI3K-related protein kinase. In support of this hypothesis, mice deficient for both *ATM* and *DNA-PK(cs)* are embryonic lethal [18]. Because *ATM* is essential for viability in flies, a temperature-sensitive *ATM* allele (*ATM*^*8*^) has been tremendously useful for investigating the mechanistic basis of adult phenotypes such as neurodegeneration [16, 17]. *ATM*^*8*^ flies contain a missense mutation that changes the final amino acid of the ATM protein from leucine to phenylalanine [15]. When raised at 25°C, *ATM*^*8*^ flies die during development, often as pupae, but lowering the temperature to 18°C largely prevents the developmental lethality. By assaying phosphorylation of the ATM substrate histone H2Av in response to ionizing radiation-induced DNA damage, we determined that ATM^8^ kinase activity is inhibited at 25°C but not at 18°C [16]. Furthermore, *ATM*^*8*^ flies that are raised at 18°C and shifted to 25°C as adults undergo progressive neurodegeneration that is caused by hyperactivation of the innate immune response in glial cells [17]. The innate immune response in flies and humans not only functions to combat pathogens but also influences the process of neuroprotection [19, 20]. In flies, the Toll and Imd (Immune deficiency) innate immune response pathways activate distinct NF-**κ**B transcription factors to control the transcription of genes that encode antimicrobial peptides (AMPs) [21]. In *ATM*^*8*^ flies, the expression of AMP genes is substantially upregulated [16, 17]. Inactivation of the Imd pathway in *ATM*^*8*^ flies by mutation of the NF-**κ**B transcription factor Relish not only reduces AMP gene expression but also blocks neuron death and increases lifespan [17]. Chronic inflammation in A-T and links between the innate immune response and neurodegeneration in disorders such as Alzheimer’s disease, Parkinson’s disease, Huntington’s disease, and Amyotrophic lateral sclerosis indicate that *ATM*^*8*^ flies are a pertinent model of neurodegeneration in A-T as well as other neurodegenerative diseases [19, 20, 22, 23].

To identify potential therapeutics for A-T and other neurodegenerative diseases, we screened a library of 2400 compounds for modifiers of the developmental lethality of *ATM*^*8*^ flies. We identified 10 compounds that reproducibly suppressed the developmental lethality of *ATM*^*8*^ flies, and we investigated the physiological and molecular effects of one of these compounds Ronnel because it paradoxically and uniquely suppressed the developmental lethality of homozygous *ATM*^*8*^ flies but enhanced the developmental lethality of heterozygous *ATM*^*8*^ flies.

## Results and discussion

### Identification of a phenotype that is sensitive to the function of ATM

Dominant modifier screens in flies have been extremely successful in identifying genes involved in specific biological processes, including genes involved in ATM-mediated neuroprotection [14]. The key feature of dominant modifier screens is a sensitized, intermediate phenotype that permits heterozygous mutations in genes that function in relevant biological processes to dominantly suppress or enhance the phenotype. Based on this paradigm, we set out to identify a sensitized, intermediate ATM-dependent phenotype where compounds that affect the function of ATM or its downstream targets would suppress or enhance the phenotype. It was previously documented that in *ATM*^*8*^ flies, ATM kinase activity and development to adulthood is temperature-sensitive [12, 15, 16]. Indeed, we found that when heterozygous *ATM*^*8*^*/TM3* flies (TM3 is a balancer chromosome that contains a wild-type *ATM* allele) were self-crossed at 25°C, no *ATM*^*8*^ F1 progeny eclosed, indicating that ATM kinase activity is below the threshold needed for viability (Table 1). In contrast, at 18°C, 24% of the F1 progeny were *ATM*^*8*^, which approaches the Mendelian ratio of 33% expected for fully viable *ATM*^*8*^ flies. Moreover, an intermediate temperature of 21°C produced an intermediate ratio of *ATM*^*8*^ F1 progeny, 16%. These data suggest that 21°C is a sensitized condition where compounds that affect ATM kinase activity or signaling through ATM pathways might alter the level of *ATM*^*8*^ progeny up to as high as 33% or down to as low as 0%.

**Table 1:** Effect of temperature on *ATM*^*8*^ viability.

Temperature	*ATM*^*8*^	Total	% *ATM*^*8*^
18°C	31	127	24
21°C	19	120	16
25°C	0	71	0

### A primary screen of 2400 compounds identifies many modifiers of *ATM*^*8*^ lethality

**Fig 1:**
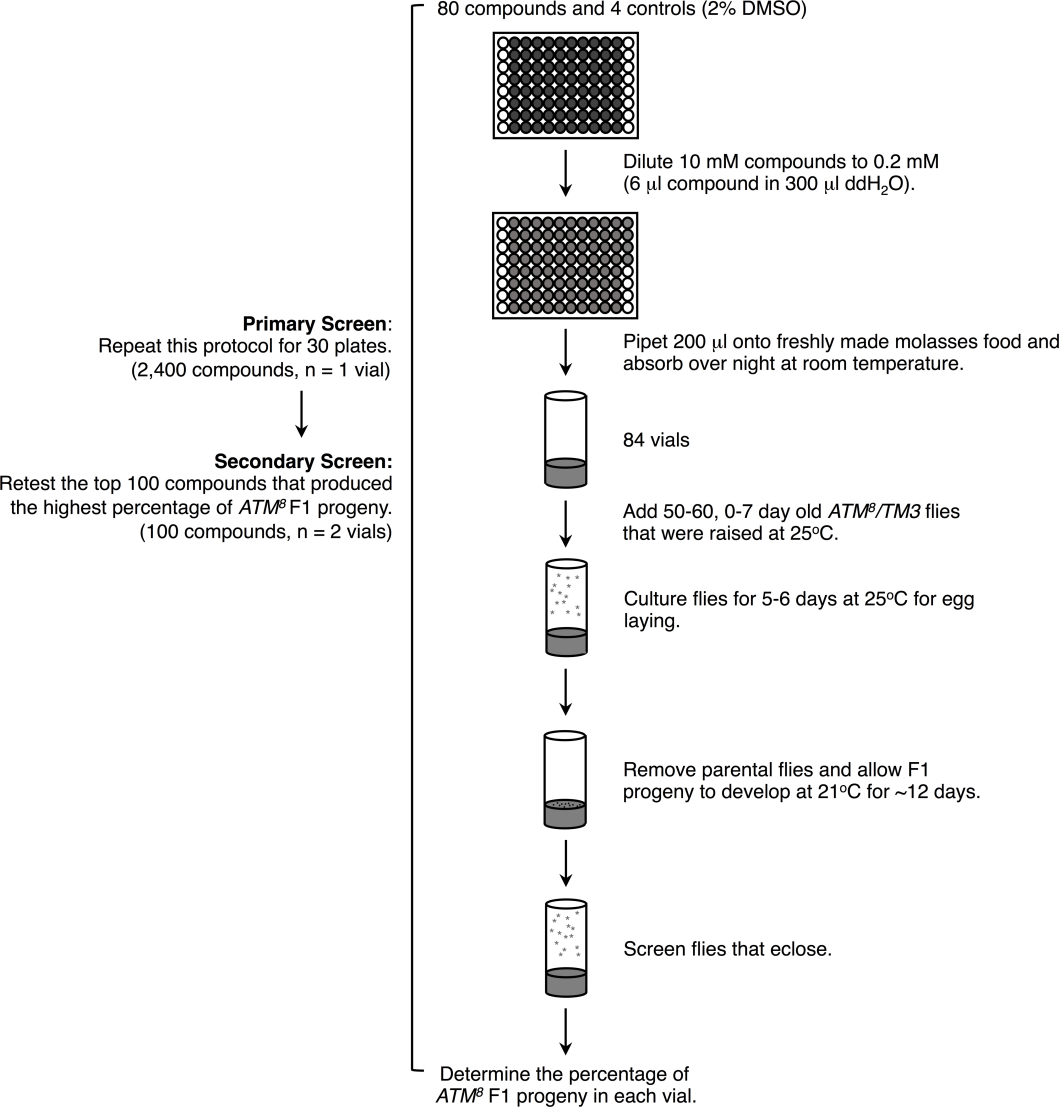
Schematic diagram of the primary and secondary screen protocols. Details are provided in the Materials and methods section as well as the Results and discussion section.

To identify compounds that affect the function of ATM, we performed a blinded screen of 2400 compounds from the Spectrum Collection (MicroSource Discovery System) for those that altered the percent eclosion of *ATM*^*8*^ flies at 21°C. The library included FDA approved drugs, natural products, and bioactive components. Details of the screening protocol are described in the Materials and methods and illustrated in Fig 1. In brief, for each compound, 200 **μ**l of 0.2 mM compound in 2% dimethyl sulfoxide (DMSO) was evenly applied to the surface of newly prepared molasses food in vials. This concentration was selected based on other screens in flies that used 0.05 or 0.1 mM compounds that were homogenously mixed in food [10, 11, 24]. 50–60 young *ATM*^*8*^*/TM3* flies were added to the vials, and the flies were cultured at 25°C for 5–6 days to allow egg-laying, at which time the parental flies were removed and the vials were transferred to 21°C for about 12 days, enough time for eggs to have developed to adults. This protocol allowed compounds to be consumed *ad libitum* by parental flies as well as F1 larvae. *ATM*^*8*^ and *ATM*^*8*^*/TM3* F1 adults were counted and the percent *ATM*^*8*^ flies was determined. In the primary screen, we only tested each of the 2400 compounds once, but, in a secondary screen, we retested the top 100 compounds two more times to identify compounds that had a reproducible effect.

Table 2 provides an overview of the findings, and S1 Table provides data for each compound. 147 compounds completely blocked the production of *ATM*^*8*^ progeny. Of these, 23 compounds, including several insecticides, also blocked the production of *ATM*^*8*^*/TM3* flies either by killing the parental flies and preventing egg-laying or by inhibiting the development of progeny. The other 124 compounds that preferentially blocked production of *ATM*^*8*^ progeny may provide insights into ATM function; however, there were no obvious commonalities among these compounds. 892 compounds had little or no effect on the development of *ATM*^*8*^ relative to *ATM*^*8*^*/TM3* flies leading to 10–20% *ATM*^*8*^ progeny, which was not substantially different than 14% *ATM*^*8*^ progeny for the control (i.e., 2% DMSO) (Table 3). 819 compounds were detrimental to the development of *ATM*^*8*^ relative to *ATM*^*8*^*/TM3* flies, reducing *ATM*^*8*^ progeny to 1–10%. In contrast, the remaining 542 compounds were beneficial to the development of *ATM*^*8*^ relative to *ATM*^*8*^*/TM3* flies, increasing *ATM*^*8*^ progeny to >20%. In summary, the primary screen served the desired purpose of broadly categorizing the collection of 2400 compounds into those that were detrimental, neutral, or beneficial to the development of *ATM*^*8*^ flies.

**Table 2:** Overview of the primary screen.

% *ATM*^*8*^	Number of compounds
0	147
1–10	819
10–20	892
20–30	425
30–40	99
40–50	16
50–60	0
60–70	1
70–80	0
80–90	0
90–100	1

**Table 3:** Top 10 compounds that reproducibly rescue *ATM*^*8*^ developmental lethality.

	Primary Screen*			Secondary Screen**		
Compound (0.2mM)	*ATM*^*8*^	Total	% *ATM*^*8*^	*ATM*^*8*^	Total	% *ATM*^*8*^
Ronnel	20	20	100	21	23	91
Estragole	17	50	34	32	126	25
Piperic acid	10	32	31	24	99	24
Quassin	16	47	34	24	100	24
Stigmasterol	10	32	31	32	153	21
Sodium thioglycolate	13	40	33	21	108	19
Avocatin B	11	33	33	22	114	19
Usinic acid	10	32	31	24	129	19
Hypoxanthine	13	40	33	27	151	18
Tyramine	13	38	34	12	117	10
Control (2% DMSO)	670	4796	14	5	441	1

*2400 compounds from the Spectrum Collection (MicroSource Discovery System)

**100 compounds from the primary screen with the highest percent *ATM*^*8*^ viability

### A secondary screen of 100 compounds identifies reproducible suppressors of *ATM*^*8*^ lethality

We performed a secondary screen of the 100 compounds that produced the highest percent *ATM*^*8*^ progeny in the primary screen (S2 Table). Each compound was tested twice under the same conditions as the primary screen. However, for unknown reasons, controls in the secondary screen had 1% rather than 14% *ATM*^*8*^ progeny observed in the primary screen (Table 3). Despite these more stringent conditions, 18 compounds substantially increased the percent *ATM*^*8*^ progeny in one of the two trials. Moreover, 10 compounds, listed in Table 3 and hereafter referred to as the top 10 compounds, substantially increased the percent *ATM*^*8*^ progeny in both trials. Thus, in three independent biological trials, the top 10 compounds reproducibly suppressed the developmental lethality due to reduced ATM kinase activity.

Three of the top 10 compounds, Ronnel (also known as fenchlorphos), estragole (also known as p-allylanisole), and stigmasterol, are inhibitors of the enzyme acetylcholinesterase (AChE), which catalyzes breakdown of the neurotransmitter acetylcholine (ACh) to choline [25–27]. Inhibition of AChE leads to accumulation of ACh at synapses and stimulation of nerves and muscles in nervous systems. AChE inhibitors have been used in the treatment of Alzheimer’s and Parkinson’s disease patients who have reduced levels of ACh in the brain [28, 29]. Thus, we retested AChE inhibitors (i.e., donepezil hydrochloride, tacrine hydrochloride, dichlorvos, galantamine, and rivastigmine tartrate) from the primary screen that were not included in the secondary screen. In the primary screen, donepezil hydrochloride, tacrine hydrochloride, and dichlorvos weakly suppressed the developmental lethality of *ATM*^*8*^ flies, but neither these nor the other AChE inhibitors affected *ATM*^*8*^ lethality in the retest (S3 Table). Similarly, four of the AChE inhibitors failed to suppress *ATM*^*8*^ lethality at other concentrations, ranging from 0.002 to 2 mM or when used in combination (S4 Table). Nevertheless, direct evaluation of AChE activity and ACh levels are needed to definitively determine the extent to which inhibition of AChE leads to suppression of *ATM*^*8*^ lethality.

Ronnel may also act through other mechanisms, as it is a member of the organophosphate class of compounds that not only inhibit AChE but also have non-cholinergic effects, including disruption of mitochondrial oxidative phosphorylation and mitochondrial membrane potential [30]. Interestingly, six of the other top 10 compounds cause mitochondrial dysfunction through a variety of mechanisms; hypoxanthine inhibits oxidative phosphorylation [31], quassin and usnic acid cause mitochondrial depolarization [32, 33], sodium thioglycolate (also known as 2-mercaptoacetate) and avocatin B inhibit mitochondrial fatty acid oxidation [34, 35], and tyramine inhibits mitochondrial respiration [36]. Thus, based on the finding that elimination of defective mitochondria by mitophagy in ATM-deficient neurons and animals has beneficial consequences [8], the identified compounds may trigger mitophagy of partially dysfunctional mitochondria in *ATM*^*8*^ cells by further reducing their function and thereby improve the mitochondrial network’s integrity and functionality.

### The top 10 compounds are allele-specific suppressors of *ATM* lethality

To investigate the mechanisms by which the top 10 compounds suppress the developmental lethality of *ATM*^*8*^ flies, we examined their effect on other recessive lethal *ATM* alleles, *ATM*^*3*^ and *ATM*^*4*^, that were identified in the same chemical mutagenesis screen that identified *ATM*^*8*^ [15]. *ATM*^*3*^ contains a nonsense mutation that truncates the ATM protein at residue 600 out of 2429, and *ATM*^*4*^ contains a missense mutation that changes leucine 284 to histidine. By genetic criteria, *ATM*^*3*^ and *ATM*^*4*^ are both null alleles. In three independent tests of each compound at 0.2 mM, we found that none of the top 10 compounds rescued the developmental lethality of even a single *ATM*^*3*^ or *ATM*^*4*^ fly. These data argue that some level of ATM activity is required for suppression of developmental lethality by the top 10 compounds.

### Ronnel is a dose-dependent suppressor of *ATM*^*8*^ and enhancer of *ATM*^*8*^*/TM3* lethality

Among the top 10 compounds, Ronnel (also known as fenchlorphos) stood out because >90% of the F1 progeny from self-crosses of *ATM*^*8*^*/TM3* flies were *ATM*^*8*^, which is considerably higher than the maximum expected 33% if both *ATM*^*8*^ and *ATM*^*8*^*/TM3* flies are fully viable (Table 3). These data indicate that Ronnel is both beneficial to the development of *ATM*^*8*^ flies and toxic to the development of *ATM*^*8*^*/TM3* flies. In support of this conclusion, the number of *ATM*^*8*^ flies that eclosed per vial with Ronnel was similar to the number for the other top 10 compounds, but the total number of eggs from which the flies developed was substantially lower because Ronnel was lethal to the parental flies (Table 3). In other words, since a similar number of *ATM*^*8*^ flies eclosed from fewer eggs, it means that *ATM*^*8*^ flies that usually would have died during development were able to survive, indicating rescue by Ronnel rather than tolerance to Ronnel. Furthermore, the effect of 0.2 mM Ronnel during fly development is dependent on the level of ATM activity; Ronnel was unable to rescue the lethality of flies with no ATM activity (i.e., *ATM*^*3*^ and *ATM*^*4*^ at 25°C), it rescued the lethality of flies with low ATM activity (i.e., *ATM*^*8*^ flies at 21°C), and it increased the lethality of flies with higher ATM activity (i.e., *ATM*^*8*^*/TM3* flies at 21°C and *ATM*^*3*^*/TM3*, *ATM*^*4*^*/TM6B*, and wild-type flies at 25°C).

To further characterize Ronnel, we used the screening protocol to examine the effect of different concentrations of Ronnel on the developmental lethality of *ATM*^*8*^ flies. As observed in the primary and secondary screens, 0.2 mM Ronnel produced >90% *ATM*^*8*^ progeny (Table 4). A 10-fold lower concentration of Ronnel (0.02 mM) reduced *ATM*^*8*^ progeny to 59% and even lower concentrations (0.002–0.00002 mM) reduced *ATM*^*8*^ progeny to 14–19%, which was similar to the 20% *ATM*^*8*^ progeny observed in DMSO controls. Fewer total progeny were produced at high concentrations of Ronnel because it killed most of the parental flies within 48 hrs thereby reducing the number of eggs laid. Moreover, the percent *ATM*^*8*^ flies at 0.02 mM Ronnel was lower than at 0.2 mM Ronnel because of increased viability of *ATM*^*8*^*/TM3* progeny rather than reduced viability of *ATM*^*8*^ progeny. These data indicate that molecular events targeted by Ronnel that suppress *ATM*^*8*^ lethality and enhance *ATM*^*8*^*/TM3* lethality are sensitive to different concentrations of Ronnel.

**Table 4:** Dose-dependent effect of Ronnel on *ATM*^*8*^ viability.

Ronnel (mM)	*ATM*^*8*^	Total	% *ATM*^*8*^
0.2	19	20	95
0.02	19	32	59
0.002	24	170	14
0.0002	27	183	15
0.00002	34	180	19
% DMSO Control (2–0.0002)	181	893	20

### Ronnel does not adversely affect the lifespan of *ATM*^*8*^ flies

**Fig 2:**
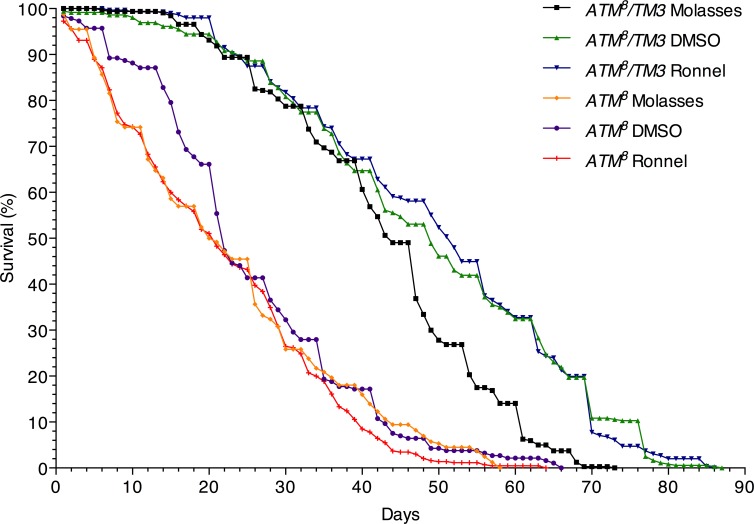
Ronnel does not affect the lifespan of *ATM*^*8*^ flies. The survival of *ATM*^*8*^ and *ATM*^*8*^*/TM3* flies raised on the indicated food source was monitored over time until all of the flies had died. *ATM*^*8*^ molasses (n = 244), *ATM*^*8*^ 0.2% DMSO (n = 186), *ATM*^*8*^ 0.02 mM Ronnel in 0.2% DMSO (n = 435), *ATM*^*8*^*/TM3* molasses (n = 320), *ATM*^*8*^*/TM3* 0.2% DMSO (n = 360), and *ATM*^*8*^*/TM3* 0.02 mM Ronnel in 0.2% DMSO (n = 296).

To investigate whether treatment with Ronnel during the development of *ATM*^*8*^ flies has long-lasting effects during adulthood, we determined the lifespan of *ATM*^*8*^ and *ATM*^*8*^*/TM3* flies at 25°C after being raised at 21°C on molasses food, molasses food with 0.2% DMSO (i.e., DMSO), or molasses food with 0.02 mM Ronnel in 0.2% DMSO (i.e., Ronnel). We used 0.02 mM rather than 0.2 mM Ronnel for this and subsequent analyses of Ronnel because it was less toxic to parental *ATM*^*8*^*/TM3* flies and *ATM*^*8*^*/TM3* progeny, which led to more *ATM*^*8*^*/TM3* flies to analyze (Table 4). This study showed that Ronnel had no effect on the lifespan of either *ATM*^*8*^ or *ATM*^*8*^*/TM3* flies (Fig 2). Kaplan-Meier analysis revealed that the mean lifespan of *ATM*^*8*^ and *ATM*^*8*^*/TM3* flies was not significantly different when raised with or without Ronnel (S5 Table). Furthermore, with and without Ronnel, *ATM*^*8*^ flies had similar mean lifespans that were significantly shorter than those of *ATM*^*8*^*/TM3* flies (P<0.01). Thus, the mechanism by which Ronnel affects the developmental of *ATM*^*8*^ and *ATM*^*8*^*/TM3* flies does not cause lasting damage or repair that affects the lifespan of adult flies.

### Ronnel does not affect the innate immune response of *ATM*^*8*^ flies

**Fig 3:**
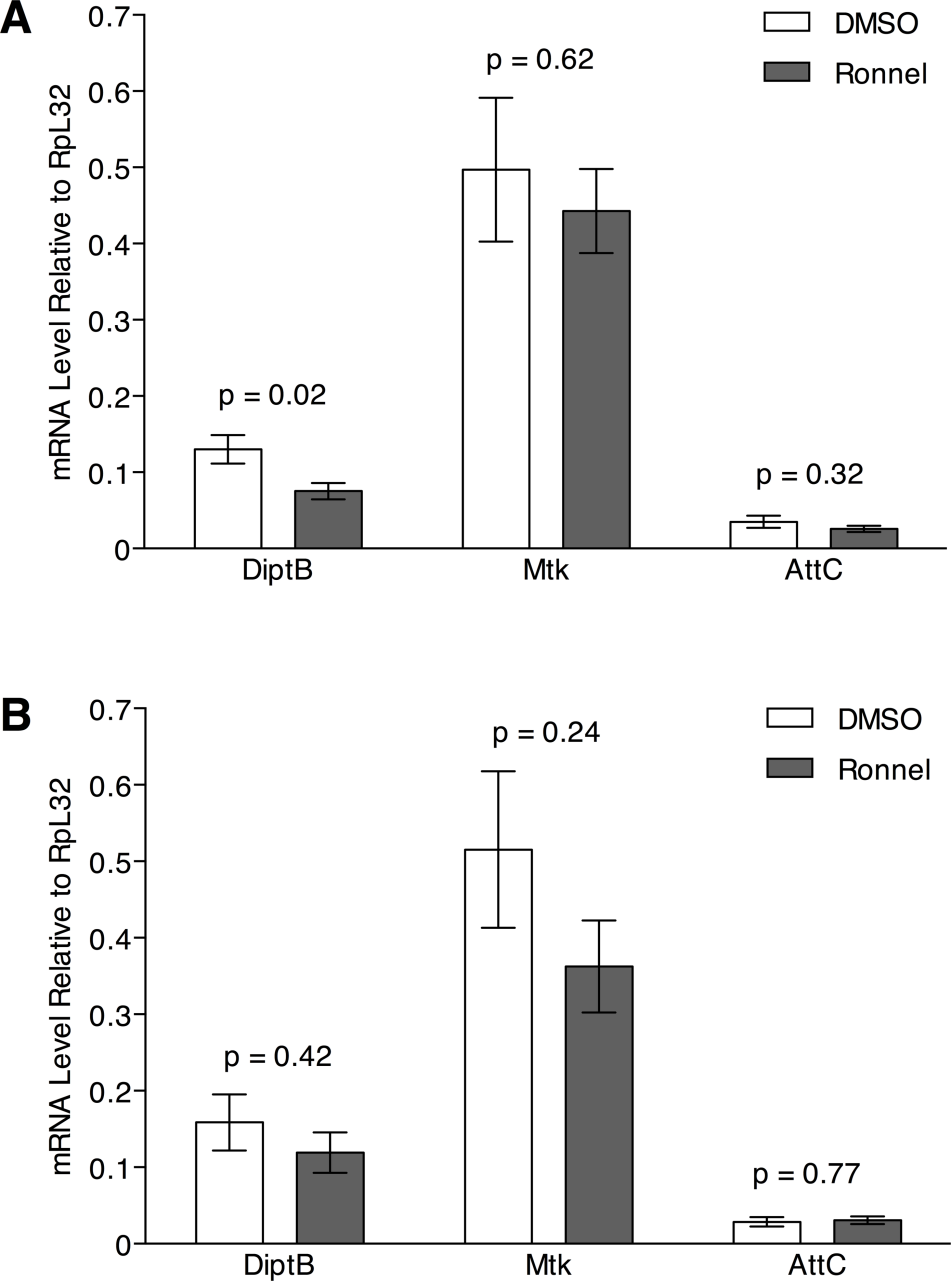
Ronnel does not affect the innate immune response of *ATM*^*8*^ flies. The expression of AMP genes (*DiptB*, *Mtk*, and *AttC*) was determined by RT-qPCR in (A) *ATM*^*8*^ flies raised on 0.2% DMSO (DMSO) or 0.02 mM Ronnel in 0.2% DMSO (Ronnel) at 21°C and transferred to 25°C for 24 hrs or (B) *ATM*^*8*^ flies raised at 18°C and transferred to 0.2% DMSO (DMSO) or 0.02 mM Ronnel in 0.2% DMSO (Ronnel) at 25°C for 24 hrs. Data are presented as the average and standard error of the mean for multiple independent samples. P-values were determined based on an unpaired equal-variance two-tail t-test.

The innate immune response is hyperactivated in glial cells of *ATM*^*8*^ flies, and genetic manipulations that reduce the innate immune response prevent neurodegeneration and increase the lifespan of *ATM*^*8*^ flies [16, 17]. Thus, Ronnel-mediated suppression of the developmental lethality of *ATM*^*8*^ flies might involve effects on the innate immune response. To test this hypothesis, we monitored activation of the innate immune response by quantifying mRNA levels of AMP genes *Diptericin B* (*DiptB*), *Metchnikowin* (*Mtk*), and *Attacin C* (*AttC*). At 24 hrs after development of *ATM*^*8*^ flies on molasses food containing 0.2% DMSO (i.e., DMSO) or 0.02 mM Ronnel in 0.2% DMSO (i.e., Ronnel), *Mtk* and *AttC* expression was equivalent between DMSO- and Ronnel-treated flies and *DiptB* expression was about 2-fold lower in Ronnel-treated flies (Fig 3A). Similarly, the expression of all three AMP genes was not affected in *ATM*^*8*^ flies fed Ronnel as adults, that is, raised on molasses food at 18°C and transferred to molasses food containing DMSO or Ronnel for 24 hrs (Fig 3B). Longer treatments of *ATM*^*8*^ adult flies with 0.02 mM Ronnel were not possible because it killed too many flies within 48 hrs. These data indicate that the innate immune response is unlikely to play a role in Ronnel-mediate suppression of the developmental lethality of *ATM*^*8*^ flies or the lethality of adult *ATM*^*8*^ flies.

### DNA damage accumulates in the brain of *ATM*^*8*^ flies

**Fig 4:**
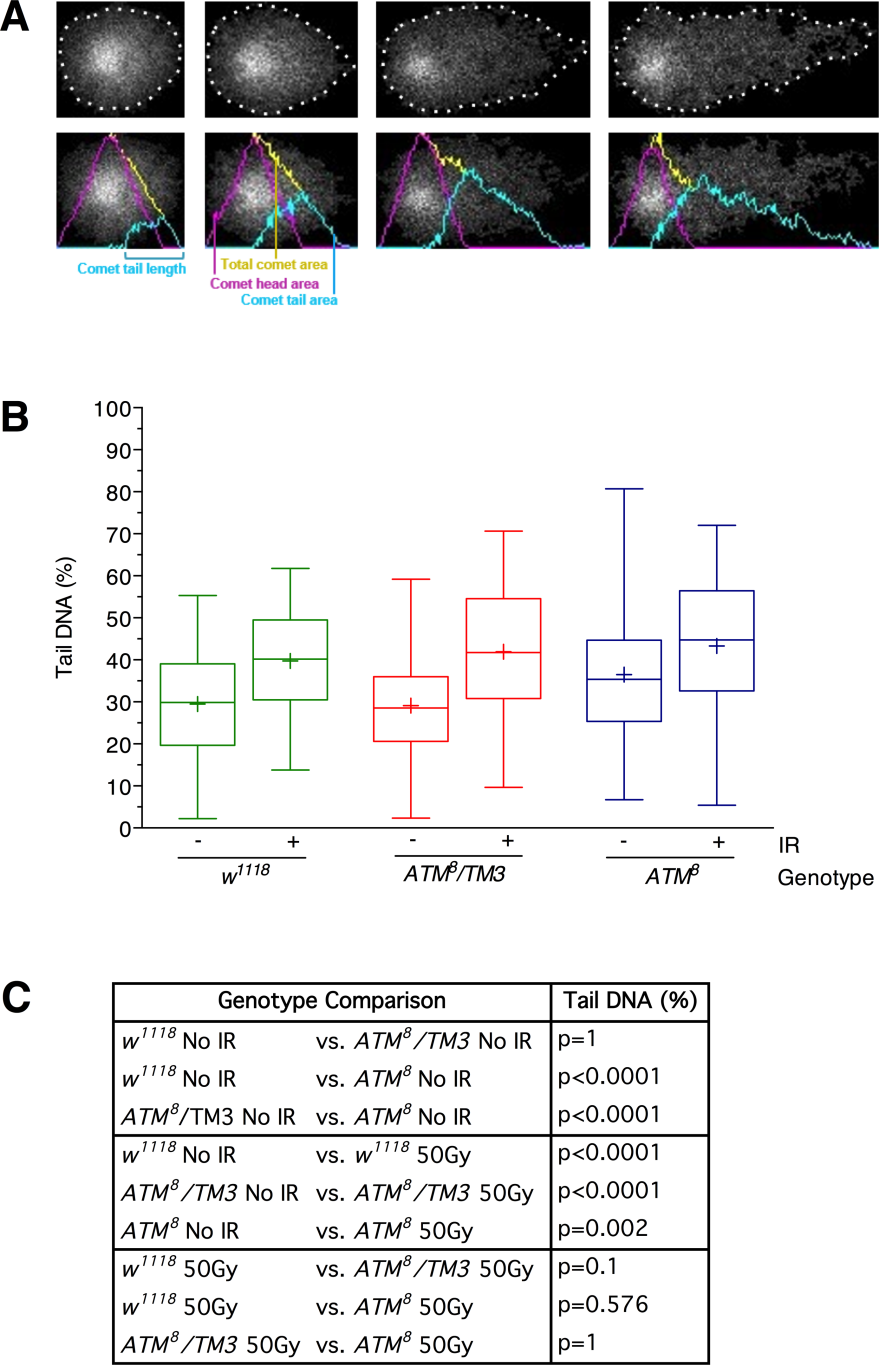
DNA damage accumulates in the brain of *ATM*^*8*^ flies. (A, top row) Microscopy images of four examples of individual cells subjected to the Comet assay. The cells are arranged left to right from low to high levels of DNA damage. Dotted lines indicate the area of electrophoresed DNA. (A, bottom row) Output from the CometScore Pro Software analysis of the cells shown in the top row. (B) The Comet assay was used to determine the percent tail DNA for brain cells of flies of the indicated genotype and either not exposed to ionizing radiation (IR) (-) or exposed to 50 Gy of IR (+). All data points were graphed using a box and whisker plot in Prism 7.0 (Graphpad) statistical software. Boxes indicate the middle 50% of the data points, lines in the middle of the boxes indicate the median, +s in the boxes indicate the mean, and the maximum and minimum whiskers indicate 95% of the data points (minimum whisker begins at 2.5% and maximum whisker ends at 97.5%). >200 comets were analyzed for each condition. (C) P-values for data presented in panel B were based on an unpaired equal-variance two-tail t-test.

The primary function ascribed to ATM is in the recognition and repair of DNA double-strand breaks [1–3]. Abnormally high levels of DNA damage have been reported in tissue culture cells and organisms, including flies, that contain *ATM* mutations, and DNA damage may be responsible for triggering neurodegeneration and other phenotypes in A-T patients [12–17]. To test the possibility that DNA damage in the brain contributes to the developmental lethality of *ATM*^*8*^ flies, we measured DNA damage using the Comet assay, which involves lysing cells in low-melt agarose and electrophoresing the released DNA [37]. Fragments of damaged DNA migrate faster than intact chromosomes during gel electrophoresis and when visualized with a fluorescent dye resemble a comet (Fig 4A). The amount of DNA in the comet tail relative to the total amount of DNA (i.e., percent tail DNA) is a measure of the amount of DNA damage. We found that cells from dissociated brains of 0–3 day old *ATM*^*8*^ flies had a significantly higher percent tail DNA than *w*^*1118*^ flies (a standard laboratory strain), whereas *ATM*^*8*^*/TM3* flies had the same percent tail DNA as *w*^*1118*^ flies (Fig 4B and 4C). Therefore, ATM activity is required in the brain to prevent the accumulation of DNA damage, and the level of ATM activity determines the extent of DNA damage.

To confirm that the Comet assay quantitatively detects DNA damage in the brain, we used it to analyze cells from dissociated brains of 3 day old flies 1 hr after exposure to 50 Gy of ionizing radiation (IR), which induces DNA double-strand breaks. In all of the flies examined, *w*^*1118*^, *ATM*^*8*^*/TM3*, and *ATM*^*8*^, IR significantly increased the percent tail DNA relative to unirradiated flies (Fig 4B and 4C). Thus, the Comet assay is able to detect a wide range DNA damage levels in brain cells.

### Ronnel exacerbates DNA damage in *ATM*^*8*^ flies

**Fig 5:**
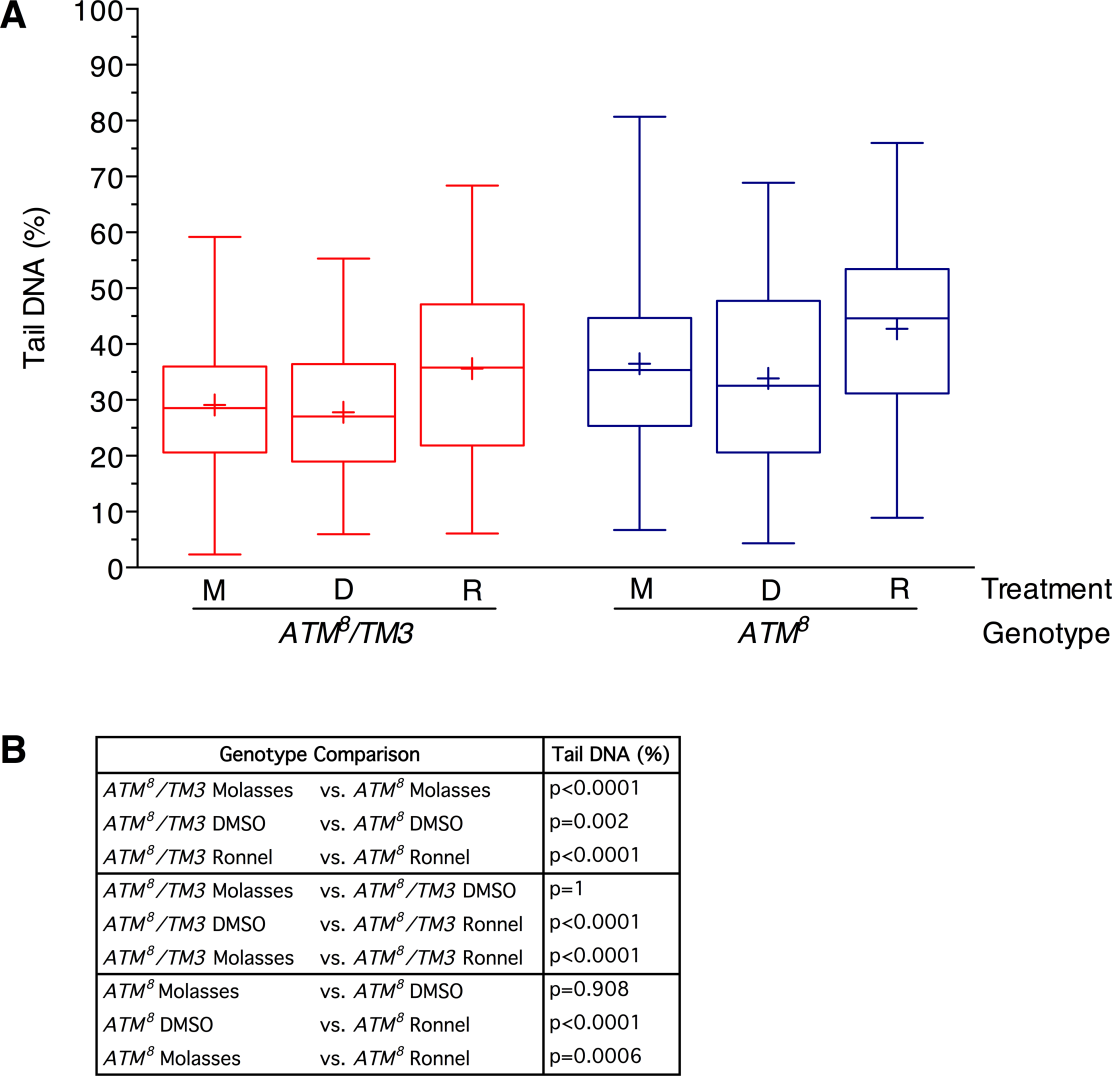
Ronnel increases the accumulation of DNA damage in *ATM*^*8*^ flies. (A) The Comet assay was used to determine the percent tail DNA for brain cells of flies of the indicated genotype and raised on molasses food (M), molasses food with 0.2% DMSO (D), or molasses food with 0.02 mM Ronnel in 0.2% DMSO (R). All data points were graphed using a box and whisker plot in Prism 7.0 (Graphpad) statistical software. Boxes indicate the middle 50% of the data points, lines in the middle of the boxes indicate the median, +s in the boxes indicate the mean, and the maximum and minimum whiskers indicate 95% of the data points (minimum whisker begins at 2.5% and maximum whisker ends at 97.5%). >200 comets were analyzed for each condition. (B) P-values for data presented in panel A were based on an unpaired equal-variance two-tail t-test.

To investigate whether Ronnel suppresses the developmental lethality of *ATM*^*8*^ flies by affecting the level of DNA damage, we used the Comet assay to determine the level of DNA damage in 0–3 day old *ATM*^*8*^ and *ATM*^*8*^*/TM3* flies that were raised at 21°C on molasses food, molasses food with 0.2% DMSO (i.e., DMSO), or molasses food with 0.02 mM Ronnel in 0.2% DMSO (i.e., Ronnel). As observed in Fig 4, *ATM*^*8*^ flies had higher percent tail DNA than *ATM*^*8*^*/TM3* flies under all of the culturing conditions (Fig 5). Moreover, relative to molasses food and DMSO, Ronnel increased the percent tail DNA in both *ATM*^*8*^ and *ATM*^*8*^*/TM3* flies, indicating that Ronnel promotes DNA damage. This is consistent with studies showing that organophosphate compounds induce DNA damage in multiple cell types [38, 39]. Since Ronnel had the same effect on DNA damage in *ATM*^*8*^ and *ATM*^*8*^*/TM3* flies but had opposite effects on the development of these flies, the effects of Ronnel on DNA damage and development are unlikely to be mechanistically related.

## Conclusions

Our pharmacological screen identified 10 compounds that suppressed the developmental lethality caused by reduced ATM kinase activity in flies (Table 3). Studies of Ronnel argue against a suppression mechanism that affects the level of the innate immune response or DNA damage (Figs 3–5). Subsets of the 10 compounds are known to inhibit the enzyme acetylcholinesterase (AChE) or the function of mitochondria, and Ronnel inhibits both AChE and mitochondria [27, 30]. These data implicate acetylcholine (ACh) in the suppression mechanism. Inhibition of AChE increases ACh levels by preventing its breakdown, and inhibition of mitochondrial function may increase ACh levels by triggering mitophagy of dysfunctional mitochondria, thereby improving the overall functionality of mitochondria, which produce acetyl-CoA, a substrate for ACh synthesis. In support of this hypothesis, AChE inhibitors have shown benefits in Alzheimer’s and Parkinson’s disease patients [28, 29], and reduced mitophagy contributes to mitochondrial dysfunction in many neurodegenerative diseases, including A-T [8, 40, 41]. However, the mechanism is likely to be complex, since some AChE inhibitors did not suppress the developmental lethality of *ATM* mutant flies (S3 and S4 Tables). Nevertheless, the pharmacological screen has uncovered specific avenues to explore for the treatment of A-T.

## Materials and methods

### Drosophila strains

Fly stocks were maintained on standard cornmeal-molasses food at 25°C, unless otherwise stated. *ATM*^*8*^*/TM3*,*Sb*, *ATM*^*3*^*/TM3*,*Sb*, and *ATM*^*4*^*/TM6B*,*Hu* flies (referred to as *ATM*^*8*^*/TM3*, *ATM*^*3*^*/TM3*, and *ATM*^*4*^*/TM6B*, respectively) were obtained from the Bloomington Drosophila Stock Center. To generate *ATM*^*8*^ flies, *ATM*^*8*^/*TM3* flies were raised at 18°C or 21°C. Heterozygous *ATM*^*8*^*/TM3* flies were maintained at 25°C.

### Pharmacological screens

2400 compounds from the Spectrum Collection (MicroSource Discovery Systems) were obtained in a 96-microplate format, 30 plates of 80 compounds (columns 1 and 12 empty), and were stored at -80°C. Each plate was thawed at room temperature in light protective packaging for 24 hrs before use. After use, all plates were flushed with nitrogen gas, immediately capped, and stored at -80°C to prevent degradation. All compounds were provided in 100% DMSO at a concentration of 10 mM. 6 **μ**l of each compound was diluted in 300 **μ**l of deionized, distilled H_2_O (ddH_2_O) to a final concentration of 0.2 mM in 2% DMSO. 200 **μ**l of 0.2 mM compound in 2% DMSO or control 2% DMSO was evenly distributed over the top of freshly made molasses food [42] in vials before the food completely solidified. Compounds were allowed to absorb into the molasses food overnight at room temperature. As described in Fig 1, one plate was tested at a time. Compounds were blindly tested through identification by plate number, row, and column position. For each plate screened, four vials of control 2% DMSO were screened. 0–7 day old *ATM*^*8*^*/TM3* flies raised at 25°C were allowed to lay eggs on molasses food with compound for 5–6 days at 25°C. *ATM*^*8*^*/TM3* parents were removed from the vials, the vials were incubated at 21°C for ~12 days, and the percent *ATM*^*8*^ progeny was determined. For the primary screen, each compound was tested once. For the secondary screen, the 100 compounds with the highest percent *ATM*^*8*^ from the primary screen were retested in duplicate.

### Lifespan assay

*ATM*^*8*^ and *ATM*^*8*^*/TM3* flies were raised on molasses food, molasses food with 200 **μ**l of 0.2% DMSO, or molasses food with 200 **μ**l 0.02 mM Ronnel in 0.2% DMSO at 21°C, collected at 0–5 days old, transferred to fresh molasses vials at approximately 20 flies per vial, and aged at 25°C. The day of collection was designated day 1. Surviving flies were counted daily until all flies had died. Flies were transferred to new vials approximately every 3 days. Lifespan assays for the different genotypes and compounds were performed at the same time. >186 flies were examined for each assay condition. The number of flies examined is provided in the legend for Fig 2. Kaplan-Meier survival analysis, standard t-tests, and chi square tests were performed using Oasis online application for survival analysis (sbi.postech.ac.kr/oasis/) and Rstudio statistical software.

### RT-qPCR analysis

To determine the effect of Ronnel on AMP gene expression when fed during development, *ATM*^*8*^ flies were raised on molasses food with 0.2% DMSO or molasses food with 0.02 mM Ronnel in 0.2% DMSO at 21°C, collected at 0–7 days post-eclosion, transferred to molasses food vials without compound or DMSO at approximately 50 flies per vial, and incubated at 25°C for 24 hrs. Total RNA was isolated from 50 whole flies for each experimental condition, *ATM*^*8*^ DMSO (n = 13) and *ATM*^*8*^ Ronnel development (n = 14). To determine the effect of Ronnel on AMP gene expression when fed to adult flies, *ATM*^*8*^ flies were raised on molasses food at 18°C, collected at 0–7 days old, transferred to fresh molasses food with 2% DMSO or molasses food with 0.02 mM Ronnel in 0.2% DMSO at approximately 50 flies per vial, and incubated at 25°C for 24 hrs. Total RNA was isolated from 50 whole flies per experimental condition, *ATM*^*8*^ DMSO (n = 15) and *ATM*^*8*^ Ronnel (n = 12). RNA was isolated using the RNeasy Plus Mini Kit (Qiagen). cDNA was generated by reverse transcription (RT) with the iScript cDNA Synthesis Kit (Bio-Rad). Real-time PCR (qPCR) was carried out as described by Katzenberger *et al*. (2006) [42]. Primer sequences for *DiptB*, *Mtk*, *AttC*, and *Rpl32* are described in Katzenberger *et al*. (2016) [42].

### Comet assay

The Comet Assay Kit (Trevigen, Catalog #4250-050K) was used with the following modifications, fly brains were dissected in cold phosphate-buffered saline (PBS) and placed in 2 ml Eppendorf tubes at a concentration of 1 brain/10 **μ**l of PBS. A minimum of 6 brains was dissected per sample. Samples were maintained on ice until they all were dissected. Samples were homogenized with plastic pestles for Eppendorf tubes, 10 **μ**l of brain homogenate was added to 75 **μ**l of LMAgarose that had been boiled and cooled to 40°C in a heat block. 50 **μ**l of LMAgarose/homogenate mix was immediately added to a CometSlide that had been warmed at 37°C. A 200 **μ**l pipette tip was used to spread the sample evenly across the sample area. Slides were placed flat, at 4°C for 30 min in the dark to cool and harden. Slides were immersed at room temperature in Lysis Solution and incubated at 4°C overnight in the dark. Slides were removed from the Lysis Solution and washed by immersion twice for 10 min in 50 ml 4°C tris-borate-EDTA (TBE). Slides were then placed in an electrophoresis unit side by side lengthwise with the white slide label at the top. The electrophoresis box was filled with 4°C TBE to just covering the sample on the slide and electrophoresed at 23V for 10 min. Excess TBE was drained from the slides, slides were washed twice for 5 min in ddH_2_O and then in 70% ethanol once for 5 min, and dried overnight at room temperature in the dark. DNA was stained by placing 100 **μ**l of SYBR Gold solution (30 ml tris-EDTA (TE) buffer and 1 **μ**l SYBR Gold (Molecular Probes, S11494)) on each circle of dried agarose for 30 min at room temperature. Excess SYBR Gold solution was drained from the slides and the slides were quickly rinsed in 50 ml ddH_2_O and dried for 20–25 min at 37°C. Comets were imaged using a Zeiss LSM 510 confocal microscope with an Imager.M1 module using a 20X/0.8 NA Plan-APOCHROMAT objective. Fluorescence was excited using an Argon laser at 488 nm. Scan mode was set to frame, frame size was 2048x2048, scan speed was 4 sec, and bit depth was 16 bit. For each sample, >200 comets were imaged and analyzed using CometScore Pro Software (TriTek Corporation). One-way ANOVA and Tukey’s post hoc test was performed using RStudio statistical software to compare percent tail DNA between pairs of samples.

### Ionizing radiation

Three day old flies were placed in empty vials at approximately 20 flies per vial. Vials were exposed to 50 Gy (5,000 rad) of gamma rays using a Cesium-137 irradiator (J. L. Sheppard Mark I unit). Brains were dissected and processed for the Comet assay 1 hr after irradiation.

## Supporting information

S1 Table Effects of 2400 compounds from the Spectrum Collection (MicroSource Discovery System) on percent eclosion of *ATM*^*8*^ flies at 21°C. (XLSX)

S2 Table A secondary screen of the 100 compounds that produced the highest percent *ATM*^*8*^ eclosion in the primary screen. (XLSX)

S3 Table A secondary screen of AChE inhibitors from the Spectrum Collection. (XLSX)

S4 Table Effects of AChE inhibitors at different concentrations and in combination. (XLSX)

S5 Table Kaplan-Meier analysis of the mean lifespan of *ATM*^*8*^ and *ATM*^*8*^*/TM3* flies raised with or without Ronnel. (XLSX)
